# Establishment and characterization of a sustained delayed-type hypersensitivity model with arthritic manifestations in C57BL/6J mice

**DOI:** 10.1186/ar3867

**Published:** 2012-06-07

**Authors:** Sara M Atkinson, Pernille A Usher, Peter H Kvist, Helle Markholst, Claus Haase, Anneline Nansen

**Affiliations:** 1 Experimental Immunology Group, Department of Immunopharmacology, Novo Nordisk A/S, Novo Nordisk Park 1, 2760 Måløv, Denmark; 2 Department of Veterinary Disease Biology, University of Copenhagen, Stigbøjlen 7, 1870 Frederiksberg, Denmark; 3 Department of Histology, Novo Nordisk A/S, Novo Nordisk Park 1 2760 Måløv, Denmark

## Abstract

**Introduction:**

Rheumatoid arthritis (RA) is a chronic progressive, inflammatory and destructive autoimmune disease, characterised by synovial joint inflammation and bone erosion. To better understand the pathophysiology and underlying immune mechanisms of RA various models of arthritis have been developed in different inbred strains of mice. Establishment of arthritis models with components of adaptive immunity in the C57BL/6J strain of mice has been difficult, and since most genetically modified mice are commonly bred on this background, there is a need to explore new ways of obtaining robust models of arthritis in this strain. This study was undertaken to establish and characterise a novel murine model of arthritis, the delayed-type hypersensitivity (DTH)-arthritis model, and evaluate whether disease can be treated with compounds currently used in the treatment of RA.

**Methods:**

DTH-arthritis was induced by eliciting a classical DTH reaction in one paw with methylated bovine serum albumin (mBSA), with the modification that a cocktail of type II collagen monoclonal antibodies was administered between the immunisation and challenge steps. Involved cell subsets and inflammatory mediators were analysed, and tissue sections evaluated histopathologically. Disease was treated prophylactically and therapeutically with compounds used in the treatment of RA.

**Results:**

We demonstrate that DTH-arthritis could be induced in C57BL/6 mice with paw swelling lasting for at least 28 days and that disease induction was dependent on CD4^+ ^cells. We show that macrophages and neutrophils were heavily involved in the observed pathology and that a clear profile of inflammatory mediators associated with these cell subsets was induced locally. In addition, inflammatory markers were observed systemically. Furthermore, we demonstrate that disease could be both prevented and treated.

**Conclusions:**

Our findings indicate that DTH-arthritis shares features with both collagen-induced arthritis (CIA) and human RA. DTH-arthritis is dependent on CD4^+ ^cells for induction and can be successfully treated with TNFα-blocking biologics and dexamethasone. On the basis of our findings we believe that the DTH-arthritis model could hold potential in the preclinical screening of novel drugs targeting RA. The model is highly reproducible and has a high incidence rate with synchronised onset and progression, which strengthens its potential.

## Introduction

Rheumatoid arthritis (RA) is a chronic systemic inflammatory disease characterized by severe synovitis leading to the destruction of articular cartilage and bone erosion. This ultimately results in joint destruction and severe disability and decreased quality of life for the affected patients [1]. Although the precise etiology and pathogenesis of RA remain unknown, several therapeutic advances have been made in recent years, most notably through blockade of tumor necrosis factor (TNF) [2,3].

To develop targeted therapeutic interventions, such as the TNF-blocking biologics currently on the market, investigators must attempt to dissect the multi-factorial nature of RA pathogenesis through the use of animal models mimicking different aspects of the disease. One animal model cannot stand alone, and in the pre-clinical screening of potential therapeutics, it is advantageous to use a range of different models that can supplement each other in the final evaluation of a drug candidate. Indeed, the predictive value of anti-rheumatic drug efficacy in pre-clinical animal models of RA is greatly enhanced if the pre-clinical efficacy testing is conducted using several different animal models [4,5].

Several animal models of arthritis exist, but none of them truly represents the human condition. Most likely each model mimics certain aspects and thus can be used as tools to increase the understanding of specific pathways of the disease. In the murine collagen-induced arthritis (CIA) model, disease is induced by immunization with type II collagen (CII) in an adjuvant. This approach leads to activation of CII-specific T cells and generation of anti-CII-specific autoantibodies and results in polyarthritis characterized by severe synovitis, cartilage destruction, and bone erosion [6,7]. Furthermore, it is possible to induce transient polyarthritis in mice on the basis of passive transfer of monoclonal anti-CII antibodies - anti-type II collagen antibody-induced arthritis (CAIA) [8,9]. The disease pathogenesis differs in the two models. Arthritis in CIA mice is a combination of a delayed-type hypersensitivity (DTH) reaction and an immune complex disease of the joint, and the disease is dependent on T and B cells [10-12]. The arthritis that develops in CAIA is a combination of immune complex disease and innate immune cell-mediated tissue destruction of the joint, in which the administered anti-CII coats the cartilage surface and leads to complement activation and Fc receptor-mediated activation of cells of the innate immune system [8,13-15]. Disease in CAIA arises independently of T and B cells. CIA susceptibility is dependent on major histocompatibility complex (MHC), and only strains carrying the H-2^q ^or H-2^r ^haplotype are susceptible. The DBA1 strain has the H-2^q ^haplotype, and male mice of this strain are the most commonly used [16]. C57BL/6 mice have the H-2^b ^haplotype and thus are considered refractory to CIA under normal circumstances [17].

In 2007, Tanaka and colleagues [18], using male BALB/c mice, described a new arthritis model - termed DTH arthritis - in which arthritis can be induced in a single paw by immunization with methylated bovine serum albumin (mBSA) combined with injection of a low dose of an anti-CII antibody cocktail (anti-CII) followed by challenge with mBSA locally in the paw. The disease is characterized by severe paw swelling, infiltration of inflammatory cells, hyperplasia of the synovial membrane, cartilage destruction, and bone erosion, and it is thought that the local T cell-mediated DTH response to mBSA in conjunction with the presence of antibodies against collagen II gives rise to the arthritic phenotype.

In this study, we aim to establish the DTH arthritis model in female C57BL/6 mice and characterize the involved cell populations and cytokines. The motivation for the transfer to this strain is the prospect of genetically modified mouse studies, which are most often on a C57BL/6 background. A robust arthritis model on a C57BL/6 background thus would be of considerable value as a tool for understanding the disease process and evaluating novel therapeutics. We confirm and extend the previous publication [18] by demonstrating that arthritic disease can be induced in C57BL/6 mice. Disease induction is dependent on CD4^+ ^cells, and macrophages, neutrophils. Osteoclasts are also heavily involved in the pathology. A clear profile of cytokines and chemokines associated with these cell subsets is induced locally in the diseased tissue. Furthermore, we demonstrate that disease can be both prevented and treated with compounds approved for the treatment of RA in the clinic.

## Materials and methods

### Animals

Female C57BL/6J mice (Taconic, Bomholtvej, Denmark) were used at 6 to 8 weeks of age. Animals were housed in a facility with a 12-hour light/dark cycle and with free access to water and standard rodent chow (Altromin^®^; Altromin Spezialfutter GmbH & Co. KG, Lage, Germany). All animal experiments have been conducted according to Danish legislation and have been approved by the Danish Animal Inspectorate and the local ethical review board.

### Induction and assessment of DTH arthritis

On day minus 7, the mice were immunized with mBSA (Sigma-Aldrich, St. Louis, MO, USA) emulsified in complete Freund's adjuvant (CFA) (Difco, Detroit, MI, USA) intradermally at the base of the tail. Four days later, they received 1,000 μg (approximately 50 mg/kg) CII mouse antibody 5-clone cocktail (Chondrex, Redmond, WA, USA) containing the clones A2-10 (IgG2a), F10-21 (IgG2a), D8-6 (IgG2a), D1-2G (IgG2b), and D2-112 (IgG2b) intravenously in 200 μL of phosphate-buffered saline (PBS). On day 0, the mice were challenged with 200 μg of mBSA subcutaneously in 20 μL of PBS in the right footpad. The left footpad was given 20 μL of PBS only and served as a control. Paw swelling was measured by using a dial thickness gage (Mitutoyo, Kanagawa, Japan) and was calculated as right paw thickness minus left paw thickness. Results are displayed as mean ± standard error of the mean (SEM). The clinical score was based on the following scoring system: 0 = normal, 1 = slight swelling of the footpad or digits, 2 = moderate swelling of the footpad or ankle or both, and 3 = severe swelling of the entire paw and ankle. Clinical score results are displayed as the median. The results are representative of at least five experiments.

### Histopathological evaluation of DTH arthritis and immunohistochemistry

Hind paws were sampled and processed by standard histopathological procedures. Briefly, paws were fixed in 4% paraformaldehyde before decalcification for 7 days in formic acid bone decalcifier (Immunocal; Decal Chemical Corporation, Tallman, NY, USA). The tissue samples were dehydrated and embedded in paraffin before sections of 3 to 5 μm were prepared and deparaffinized. For histopathological evaluation, sections were stained by using a conventional hematoxylin and eosin (H&E) tissue stain. For detection of osteoclasts, a histochemical stain for the osteoclast enzyme tartrate-resistant acid phosphatase (TRAP) was performed. Deparaffinized sections were stained in a TRAP staining solution containing 1.35 mmol naphtol AS-MX phosphate (Sigma-Aldrich), 0.362 mmol N, N-dimethylformamide (Fluka; Sigma-Aldrich), 3.88 mmol Fast Red TR salt (Sigma-Aldrich), 0.5 mmol manganese chloride (Sigma-Aldrich), and 25 mmol sodium tartrate (Sigma-Aldrich) in 0.1 M Tris buffer and then were counterstained with hematoxylin. Cell nuclei appeared blue, and the osteoclast cytoplasm appeared red. For evaluation of cartilage destruction, the Safranin O staining protocol, which stains cartilage proteoglycan red, was used. Briefly, the deparaffinized sections were first counterstained with hematoxylin before staining in 0.1% Safranin O (BDH Chemicals, Poole, UK) in distilled water followed by staining in 0.1% Fast Green (Sigma-Aldrich) in distilled water to provide the green tissue contrast. For immunohistochemical detection of F4/80^+^, monoclonal rat anti-mouse F4/80 (Abcam, Cambridge, UK) was used, and rat IgG2b (BD Pharmingen, San Diego, CA, USA) was used as isotype control. These were diluted 1:250 and incubated with the tissue sections overnight at 4°C. Prior to incubation, antigen was retrieved by using Proteinase K (Roche, Basel, Switzerland). Detection was carried out with rabbit anti-rat antibodies (Dako, Glostrup, Denmark) followed by incubation in EnVision+ System horseradish peroxidase-labeled polymer anti-rabbit antibody (Dako). To visualize the target, sections were treated with 3-3'-diamino-benzidine-tetrahydrochloride (Sigma-Aldrich) for 5 minutes. The sections were all digitally scanned and studied by using a NanoZoomer Digital Pathology Virtual Slide Viewer (Hamamatsu Photonic, Shizuoka, Japan).

### Histopathological scoring of DTH arthritis paws

Pathological changes in the paws were assessed on sections stained with H&E, TRAP, and Safranin O. The extra-articular infiltration of inflammatory cells (assessed on a scale of 0 to 3) and arthritic changes were assessed separately. Arthritic changes were assessed on metatarsal and tarsal joints, where synovitis, cartilage destruction, and bone erosion were scored separately on a scale of 0 to 3. For each of the three parameters of arthritic changes, an average between the two joint areas was calculated. In addition, new bone formation overall in the paw was scored on a scale of 0 to 3. The histology sum score was calculated by adding the five scores (extra-articular infiltration, synovitis, cartilage destruction, bone erosion, and bone formation), whereas the extra-articular infiltration score is left out in the arthritis score.

### Antigen-specific proliferation assay

Popliteal lymph nodes from the antigen- and PBS-challenged side from animals sacrificed on day 4 after DTH-arthritis induction were isolated and passed through a 70-μm cell strainer to produce single-cell suspensions and placed in complete medium containing RPMI 1640 (Invitrogen Corporation, Carlsbad, CA, USA) with 1.5% C57BL/6 mouse serum (Innovative Research, Novi, MI, USA), 1% penicillin/streptomycin (Invitrogen Corporation), and 50 μM 2-mercaptoethanol (Invitrogen Corporation). Lymph nodes draining the PBS-challenged side were pooled. Two hundred thousand cells per well were transferred to a 96-well plate, and 0, 5, 10, or 20 μg/mL mBSA (Sigma-Aldrich) was added. The plate was incubated at 37°C for 72 hours before the addition of 0.5 μCi/well ^3^H-thymidine (Amersham, now part of GE Healthcare, Little Chalfont, Buckinghamshire, UK) and incubation overnight. Results are displayed as mean ± SEM counts per minute with background subtracted. The experiment was performed once.

### Enzyme-linked immunosorbent assay

Levels of serum amyloid P component (SAP) were measured in serum from mice with DTH arthritis by using sandwich enzyme-linked immunosorbent assay (ELISA) kits (GenWay Biotech, Inc., San Diego, CA, USA) in accordance with the instructions of the manufacturer. Levels of interleukin 6 (IL-6) and total (activated + proenzyme) matrix metalloproteinase 3 (MMP3) were measured in serum from mice with DTH arthritis by using sandwich ELISA kits (R&D Systems, Minneapolis, MN, USA) in accordance with the instructions of the manufacturer. Results are displayed as mean ± SEM.

### Analysis of cell subsets in the inflammatory exudates

Cells were isolated from antigen-challenged hind paws from mice sacrificed on days 2 and 7 after DTH-arthritis induction by incubating paw tissue for 1 hour in 50 U/mL collagenase (Sigma-Aldrich) and 50 U/mL DNAse (Roche) followed by washing in PBS and counting. Cells were stained with Fixable Near IR Vital dye (Invitrogen Corporation), CD45-E-fluor 450, clone 30.F11 (eBioscience, San Diego, CA, USA), TCRβ-PerCP Cy5.5, clone H57-597 (eBioscience), CD4-Qdot 605, clone RM4-5 (Invitrogen Corporation), CD19-PE-Cy7, clone 1D3 (BD Biosciences, San Jose, CA, USA), CD11c-APC, clone HL3 (BD Biosciences), CD11b-AF700, clone M1/70 (eBioscience), F4/80-FITC, BM8 (eBioscience), and Ly6G-PE, clone 1A8 (BD Biosciences) and analyzed by using the LSRII flow cytometer and FACSDiva software version 6.1.3 (BD Biosciences). Cells were gated on singlets, live cells, and CD45^+ ^cells before individual subsets were defined. Results are displayed as mean ± SEM for fractions and as median for absolute cell counts and are representative of three experiments.

### Depletion of CD4^+ ^and CD8^+ ^cells

A dose of 1,000 μg of depleting monoclonal antibody (mAb) against CD4 (clone GK1.5; BioXcell, West Lebanon, NH, USA) or CD8 (clone 53.6.72; BioXcell) in 200 μL of PBS or 200 μL of PBS alone was administered 24 hours prior to immunization (day minus 8) and 24 hours prior to challenge (day minus 1). Depletion was confirmed by flow cytometry analysis of whole-blood samples taken 24 hours after the first depletion treatment (day -7) and on day 9 after DTH-arthritis induction. The blood was stained with CD8-FITC (clone 2.43; Santa Cruz Biotechnology, Inc., Santa Cruz, CA, USA), CD4-PE (clone RM4-4; eBioscience), CD45-eFlour450 (eBioscience), and TcRβ-APC-eFluor780 (eBioscience). Samples were analyzed as described above. Results are displayed as mean ± SEM, and the experiment was performed once.

### Multiplex analysis of cytokines in paw homogenate

Hind paws from mice with DTH arthritis were sampled at selected times after DTH-arthritis induction and each was placed in 1.25 mL of an ice-cold homogenization buffer containing 49.995 mL of 0.9% saline, one tablet of complete ethylenediaminetetraacetic acid (EDTA)-free protease inhibitor cocktail (Roche), and 5 μL of Triton X-100 (Sigma-Aldrich). The paws were homogenized by using a T25 Ultraturrax homogeniser (IKA, Staufen, Germany) followed by centrifugation at 10,000*g *for 15 minutes. The supernatants were decanted and centrifuged once more at 10,000*g *for 15 minutes. The resulting supernatants were analyzed neat for levels of TNFα, IL-1β, IL-6, IL-17, IL-12(p40), CXCL10, CXCL2, CCL2, interferon-gamma (IFNγ), and receptor activator of nuclear factor kappa-B ligand (RANKL) by using bead-based Luminex^® ^xMAP^® ^technology with Milliplex kits from EMD Millipore Corporation (Billerica, MA, USA) in accordance with the instructions of the manufacturer. Results are displayed as the median, and difference from the control value was tested. In the analysis of cytokine production in untreated animals, left PBS-challenged hind paws removed on day 14 after DTH-arthritis induction were used as controls, and the results are representative of two experiments. In the analysis of cytokine production in anti-TNFα mAb-treated or isotype control antibody-treated animals, the contralateral left PBS-challenged hind paw was used as control, and the analysis was performed once. Any values below the detection limit were set to the detection limit for the analyte in question, and any values above the detection limit were set to the upper detection limit for the analyte in question.

### TNFα blockade *in vivo*

Mice were treated with rat anti-mouse TNFα mAbs (clone XT3.11; BioXcell) or rat IgG1 (HRPN; BioXcell) in doses of 250 μg per mouse (approximately 12.5 mg/kg) in 200 μL of PBS intraperitoneally every 48 hours beginning at the time of immunization (prophylactic treatment) or the time of challenge (treatment at onset) and continuing until the end of the study (day 10 after DTH-arthritis induction). For treatment with etanercept (Enbrel™; Pfizer, Groton, CT, USA), the mice received doses of 500 μg per mouse (approximately 25 mg/kg) or 1,000 μg per mouse (approximately 50 mg/kg) in 200 μL of saline intraperitoneally every 48 hours from day 1 after DTH-arthritis induction until day 11 after DTH-arthritis induction, at which time the study was terminated. Control groups received humanized anti-trinitrophenol (TNP) IgG1 (anti-TNP hzIgG1; Novo Nordisk A/S, Måløv, Denmark) in accordance with the same protocol. An additional vehicle-treated group was included and received 200 μL of saline intraperitoneally every 48 hours from the time of DTH-arthritis induction. Results are displayed as mean ± SEM. The results of TNFα blockade are representative of several experiments, whereas the therapeutic etanercept treatment study was performed once.

### Dexamethasone treatment

Mice were treated daily with dexamethasone (Intervet, Milton Keynes, UK) in doses of 1 mg/kg in 200 μL of saline beginning at the time of DTH-arthritis onset (day 0) and continuing until day 11 after DTH-arthritis induction, at which time the study was terminated. Control groups received no treatment. Results are displayed as mean ± SEM, and the experiment was performed once.

### Statistics

Statistical analyses were conducted by using GraphPad Prism software version 5.01 (GraphPad Software, Inc., La Jolla, CA, USA). Non-parametric data or non-normal parametric data were analyzed by using the Mann-Whitney *U *test, and parametric data were analyzed by using a two-sided unpaired *t *test. For statistical analysis of the histological sum scores, an unpaired Student *t *test with Welch's correction was used. Differences between groups were considered significant when *P *values were not more than 0.05, and levels of significance were assigned as *P ≤ 0.05, **P ≤ 0.01, and ***P ≤ 0.001.

## Results

### Establishment of DTH arthritis in C57BL/6J mice

**Figure 1:**
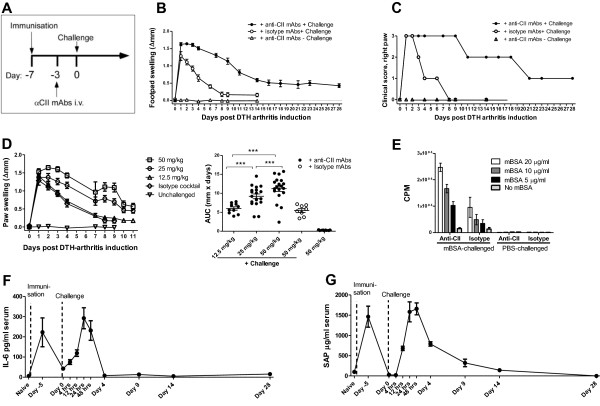
**Establishment and assessment of the delayed-type hypersensitivity (DTH)-arthritis model**. **(a) **Protocol for induction of DTH arthritis in mice. **(b) **Paw swelling (calculated as Δpaw swelling = left paw swelling - right paw swelling) on days 0 to 28 after antigen (Ag) challenge for the group that received anti-type II collagen antibody cocktail (anti-CII) + challenge. For the other two groups, paw swelling until day 14 after arthritis induction is shown. Mean ± standard error of the mean (SEM) is displayed (*n *= 9 per group). **(c) **Clinical score of the Ag-challenged paw on days 0 to 28 after Ag challenge for the group that received anti-CII + challenge. For the other two groups, area under the curve (AUC) of the paw swelling until day 14 after arthritis induction is shown. The median is shown (*n *= 9 per group). **(d) **Titration of anti-CII. AUC for days 0 to 11 after DTH-arthritis induction is shown for the groups receiving anti-CII + challenge. For the group receiving anti-CII and no challenge and the group receiving isotype cocktail and challenge, AUC for days 0 to 9 after DTH-arthritis induction is shown. Mean ± SEM is shown (*n *= 6 to 19 per group). **(e) **Ag-specific proliferation assay of cells isolated from the popliteal lymph node draining either the Ag-challenged or the phosphate-buffered saline (PBS)-challenged paw from mice receiving either anti-CII or isotype cocktail. Counts per minute (CPM) relative to the degree of ^3^H incorporation are shown with mean ± SEM (*n *= 3) in triplicate wells. **(f) **Serum levels of interleukin-6 (IL-6) on selected time points after DTH-arthritis induction. Mean ± SEM is shown (*n *= 5 per time point). **(g) **Serum levels of acute-phase protein serum amyloid P component (SAP) on selected time points after DTH-arthritis induction. Mean ± SEM is shown (*n *= 5 per time point). ***P ≤ 0.001. αCII, anti-type II collagen monoclonal antibody cocktail; i.v., intravenous; mAb, monoclonal antibody; mBSA, methylated bovine serum albumin.

The DTH-arthritis model was first described by Tanaka and colleagues in male BALB/c mice in 2007 [18]. We aimed to transfer the model to female C57BL/6J mice by using the experimental protocol outlined in Figure 1a. Briefly, the mice were immunized on day -7 with mBSA emulsified in CFA; on day -3, they were given a cocktail of anti-type II collagen antibodies (anti-CII), or an isotype control cocktail, before challenge on day 0 with mBSA in the right hind paw and vehicle (PBS) in the left hind paw. The findings by Tanaka and colleagues were confirmed by us in female BALB/c mice (data not shown), and the model was transferred with success to female C57BL/6J mice, in which paw swelling persisted for at least 28 days (Figure 1b, c). Disease onset was simultaneous, incidence was close to 100%, and inflammation was restricted to the mBSA-challenged paw. The control group receiving isotype control mAb cocktail developed a transient response consistent with a DTH response, whereas the unchallenged control group failed to develop a response. The unimmunized control group developed a very slight response on day 1 after the challenge because of the local irritant effect of mBSA (data not shown). A titration study was undertaken to determine the optimal sub-arthritogenic dose of anti-CII for elicitation of a robust and sustained paw inflammation without any signs of arthritis unrelated to challenge with mBSA, such as swelling in any paw other than the mBSA-challenged one or any paw swelling before the time of mBSA challenge. The doses tested were 12.5, 25, 50, and 75 mg/kg; 75 mg/kg anti-CII was ruled out due to the appearance of paw swelling before the time of mBSA challenge in three out of 10 animals (data not shown) and thus was not deemed to be a sub-arthritogenic dose. A dose of 50 mg/kg anti-CII resulted in the most robust paw inflammation (Figure 1d) and no sign of swelling in the absence of mBSA challenge, so this dose was chosen for use in all subsequent studies. To determine whether the response was truly restricted to the mBSA-challenged side of the animal, we performed a ^3^H-thymidine incorporation assay. This allowed us to quantify cell proliferation in response to mBSA re-stimulation on cells isolated from the popliteal lymph nodes excised from the antigen-challenged and PBS-challenged sides of the animals. The results (Figure 1e) clearly demonstrate that the proliferative response to mBSA was restricted to the lymph node draining the mBSA-challenged footpad in animals receiving either anti-CII or isotype control antibody cocktails. To have a secondary readout parameter and to determine whether DTH arthritis has a systemic disease component, serum levels of the acute-phase protein SAP and the cytokine IL-6 were measured. SAP (Figure 1g) and IL-6 (Figure 1f) levels in serum followed a biphasic time course and peaked twice: 48 hours after immunization and 24 hours (IL-6) or 48 hours (SAP) hours after the challenge. After that, the levels of SAP remained elevated above baseline until day 9 after arthritis induction. IL-6 levels had returned to baseline levels by day 4 after arthritis induction. The biphasic response corresponds to the two interventions: immunization and challenge; the use of CFA in the immunization step probably had an effect on the first observed peak in SAP and IL-6 levels.

**Figure 2:**
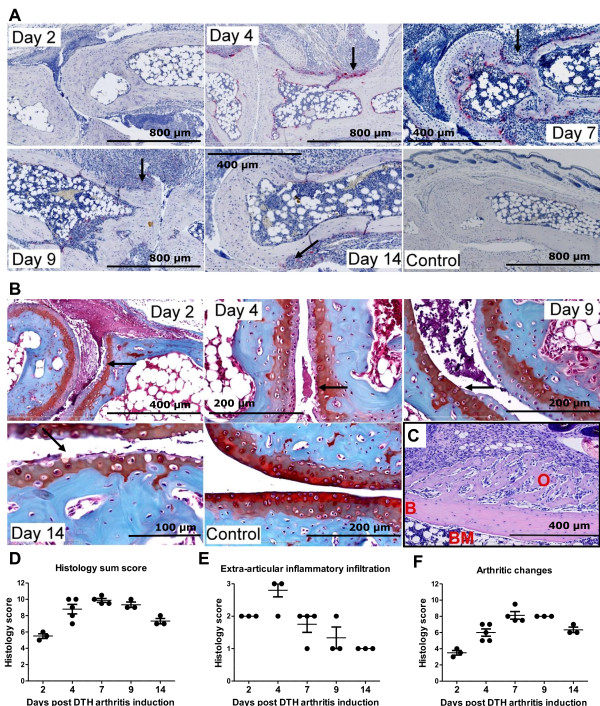
**Development of severe arthritis in mice with delayed-type hypersensitivity (DTH) arthritis**. **(a) **Histochemical stains for the osteoclast-specific enzyme tartrate-resistant acid phosphatase (TRAP). Osteoclasts appear red. Arrows indicate areas with increased osteoclast numbers and bone erosion. **(b) **Histochemical stain using the Safranin O protocol. Cartilage proteoglycan is stained red, and the intensity of the red color is proportional to the proteoglycan content of cartilage. Arrows indicate areas displaying cartilage loss, which is most prominent in the uncalcified cartilage. Mice with DTH arthritis developed severe arthritis characterized by increased cartilage degradation (as assessed by loss of Safranin O staining) and osteoclast activity (as assessed by the increase in TRAP-positive cells) and bone erosion; synovitis and pannus formation is also observed. The sections shown in (a) and (b) are representative of mice with DTH arthritis (immunization, anti-type II collagen antibody cocktail (anti-CII), and antigen challenge) and of mice receiving immunization, anti-CII, and no challenge (control). Samples were taken on days 2, 4, 7, 9, and 14. **(c) **Osteophyte shown on day 14 after DTH-arthritis induction. Hematoxylin and eosin staining was used. B, bone; BM, bone marrow; O, osteophyte. **(d) **Total sum score of histopathological changes from days 2 to 14 after DTH-arthritis induction. Maximum possible score is 15. Mean ± standard error of the mean (SEM) is shown (*n *= 3 to 5). **(e) **The score for extra-articular inflammatory infiltration from days 2 to 14 after DTH-arthritis induction. Maximum possible score is 3. Mean ± SEM is shown (*n *= 3 to 5). **(f) **Sum score of arthritic changes (calculated as total sum score of histopathological changes from which the score for extra-articular inflammatory infiltration has been subtracted) from days 2 to 14 after DTH-arthritis induction. Maximum possible score is 12. Mean ± SEM is shown (*n *= 3 to 5). Details of the histopathological scoring system can be found in the Materials and methods section.

**Figure 3:**
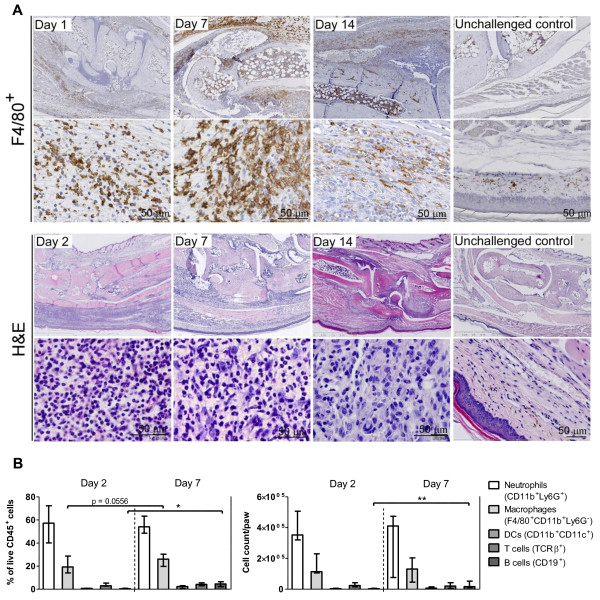
**Composition of the inflammatory infiltrate in delayed-type hypersensitivity (DTH) arthritis**. **(a) **Representative immunohistochemical stains for F4/80^+ ^cells and hematoxylin and eosin (H&E) stains from mice receiving immunization, anti-type II collagen antibody cocktail (anti-CII), and challenge. Samples were taken on days 1 (F4/80^+^), 2 (H&E), 7, and 14 after DTH-arthritis induction. Unchallenged control samples are from mice receiving immunization, anti-CII, and no challenge. Mice with DTH arthritis displayed severe inflammation characterized by an infiltration of neutrophils and F4/80^+ ^cells into the soft tissue and intra-articular space; neutrophils dominated in the early stages and in the intra-articular space. Severe hyperplasia of the synovial membrane and pannus formation were also observed. **(b) **Flow cytometric analysis of inflammatory infiltrate isolated from inflamed paws. Cell subsets are displayed both as fraction of total live CD45^+ ^cells and as absolute numbers per paw. Cells were gated on singlets, live cells, and CD45^+^, and cell subsets were defined as follows: neutrophils: Ly6G^+^CD11b^intermediate-high^; macrophages: F4/80^+^CD11b^+^Ly6G^-^; dendritic cells (DCs): CD11b^+^CD11c^+^; T cells: TcRβ^+^; B cells: CD19^+^. Median ± range is shown (*n *= 5). *P ≤ 0.05, **P ≤ 0.01. TcRβ^+^, T-cell receptor-beta-positive.

Histopathological analyses were performed to evaluate the arthritic pathology in the DTH-arthritis model, and a scoring system was applied (see Materials and methods for details). The evaluation revealed that, in the mBSA-challenged paw, the mice developed a severe arthritis and peri-articular inflammation characterized by influx of inflammatory cells, hyperplasia of the synovial membrane and pannus formation with the presence of fibroblast-like cells, increased (compared with unchallenged control animals) osteoclast activity (assessed by the increase in number of TRAP-positive cells, stained red, Figure 2a) and bone erosion, and cartilage destruction evidenced by loss of Safranin O staining (Figure 2b). The arthritis was localized in the ankle, tarsus, and metatarsophalangeal joints of the mBSA-challenged paw. Bone erosion was evident on day 4 after DTH-arthritis induction and afterwards, and cartilage destruction (assessed by loss of Safranin O staining of articular cartilage) was evident on day 2 after DTH-arthritis induction and afterwards. Osteoclast number was reduced by day 14 after DTH-arthritis induction compared with days 4, 7, and 9, but no repair of the damage to bone integrity was apparent. However, new bone formation was observed and was most prevalent as osteophytes adjacent to the affected joints of the paw (Figure 2c). The histology sum score reached a peak on day 7 after arthritis induction (Figure 2d), and the extra-articular inflammation peaked on day 4 after arthritis induction (Figure 2e). The sum score for arthritic changes (calculated as the total sum score minus the score for extra-articular inflammation) reached its peak on days 7 to 9 after arthritis induction (Figure 2f). Neutrophils comprised a large proportion of the infiltrating inflammatory cells, in both the synovial tissue and intra-articular space on days 1 and 2 after the challenge. The infiltration persisted on day 7 but was reduced by day 14 (Figure 3a). Increase in number of macrophages (F4/80^+ ^cells) was observed after the increase in neutrophils in the tissue. Macrophages were observed in great number in the soft tissue and to a lesser degree in the intra-articular space (Figure 3a). Fibroblast-like cells were also observable within the hyperplastic synovial lining and pannus tissue (Figures 2a and 3a, b), where they most likely also contribute to the inflammatory process.

These data, taken together, demonstrated that mice with DTH arthritis developed a semi-chronic arthritic disease limited to the mBSA-challenged paw and draining lymph node while still displaying a systemic component. The arthritis pathology observed included synovitis, hyperplasia of the synovial membrane and pannus formation, increased osteoclast activity, bone erosion and remodeling, and cartilage destruction.

### Inflammatory cell subset involvement

**Figure 4:**
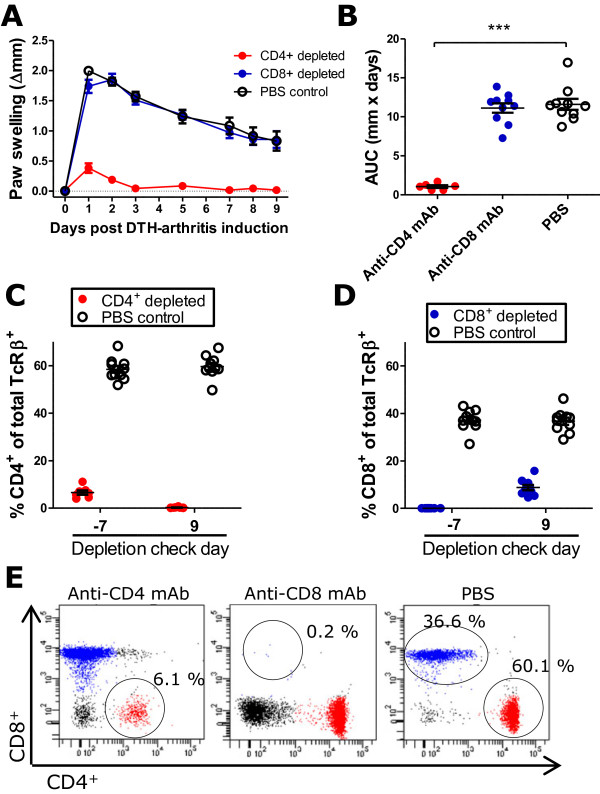
**The delayed-type hypersensitivity (DTH)-arthritis inflammatory response is dependent on CD4^+ ^T cells**. The response is dependent on CD4^+ ^cells and independent of CD8^+ ^cells, as demonstrated by the failure of mice depleted of CD4^+ ^cells to develop a paw swelling response and the ability of mice depleted of CD8^+ ^cells to develop a paw swelling response no different than that of the phosphate-buffered saline (PBS)-treated control group. **(a) **Paw swelling measured over days 0 to 9 after DTH-arthritis induction. Mean ± standard error of the mean (SEM) is shown (*n *= 6 to 10 per group). **(b) **Area under the curve (AUC) of paw swelling based on paw swelling data from days 0 to 9 after arthritis induction. Mean ± SEM is shown (*n *= 6 to 10 per group). ***P ≤ 0.001. **(c) **Percentage of CD4^+ ^cells of total T-cell receptor-beta-positive (TCRβ^+^) cells measured on days -7 and 9 after DTH-arthritis induction shown for mice receiving anti-CD4 monoclonal antibody (mAb) or PBS on days -8 and -1. The data show that CD4^+ ^cells were efficiently depleted. Cells were gated on CD45 and TCRβ. Mean ± SEM is shown (*n *= 6 to 10 per group). **(d) **Percentage of CD8^+ ^cells of total TCRβ^+ ^cells measured on days -7 and 9 after DTH-arthritis induction shown for mice receiving anti-CD8 mAb or PBS on days -8 and -1. The data show that CD8^+ ^cells were efficiently depleted. Cells were gated on CD45 and TCRβ. Mean ± SEM is shown (*n *= 6 to 10 per group). **(e) **Representative flow cytometry plots from the depletion check on day -7 demonstrating the efficiency of the depleting mAbs. Cells were gated on CD45 and TCRβ. The circled populations represent the remaining percentage of either CD4^+ ^or CD8^+ ^cells of total TCRβ^+ ^cells.

We next wanted to characterize the cell subsets involved in the inflammatory response. The histological findings described in the previous section were confirmed by flow cytometry (Figure 3b), which showed an unchanged neutrophil fraction on day 7 compared with day 2 after DTH-arthritis induction and a fraction of F4/80^+ ^cells that tended to increase from days 2 to 7 after DTH-arthritis induction. In addition, the fraction of CD19^+ ^cells increased significantly from days 2 to 7 after DTH-arthritis induction. The same picture was seen in the absolute cell counts; however, there was no observable increase in macrophage numbers. The involvement of effector T-cell subsets was investigated by treating mice with depleting mAbs against CD4 or CD8 24 hours prior to immunization and again 24 hours prior to induction of arthritis. The control group received PBS injections. Depletion of CD4^+ ^cells resulted in an abrogated paw swelling response in comparison with the PBS-treated control group, whereas there was no effect of depleting CD8^+ ^cells (Figure 4a, b). Depletion was confirmed by flow cytometry (Figure 4c-e), and although a small fraction of CD4^+ ^T cells remained on day -7, this was not sufficient to induce an inflammatory response, and by day 9 after DTH-arthritis induction, all CD4^+ ^T cells were depleted from the system. Together, these data demonstrated that neutrophils were involved in the acute stage of DTH arthritis, and the major increase in macrophage numbers occurred after a few days. In addition, development of DTH arthritis was dependent on CD4^+ ^T cells.

**Figure 5:**
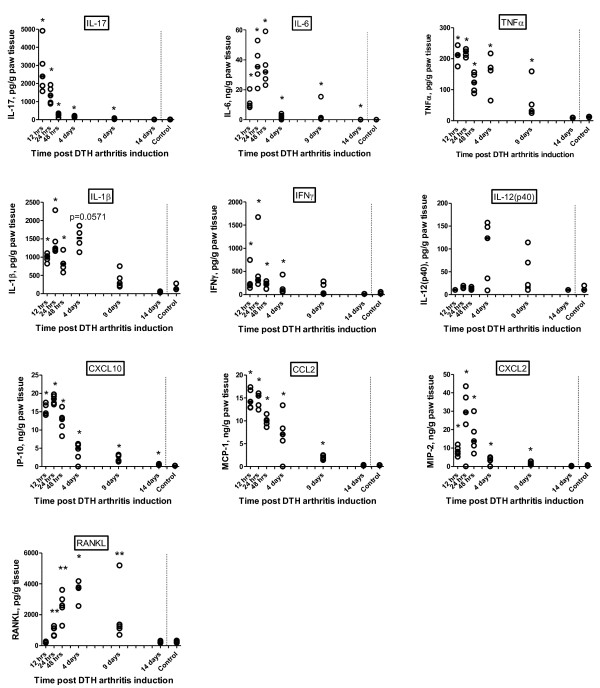
**Local profile of inflammatory mediators in the delayed-type hypersensitivity (DTH)-arthritis response**. Levels were measured by multiplex bead-based luminex analysis on homogenate supernatants from whole paws taken at 12, 24, and 48 hours and 4, 9, and 14 days after induction of DTH arthritis (*n *= 5 per time point). The line represents the median. *P ≤ 0.05, **P ≤ 0.01, compared with control value. CXCL, chemokine (C-X-C motif) ligand; IFNγ, interferon-gamma; IL, interleukin; IP-10, interferon-gamma inducible protein 10; MCP-1, monocyte chemo attractant protein 1; RANKL, receptor activator of nuclear factor kappa-B ligand; TNFα, tumor necrosis factor-alpha.

Since the dominant effector cell subsets locally in the inflamed paw had been identified, it was interesting to analyze the profile of inflammatory mediators produced locally in the arthritic paw and their temporal pattern. The results (Figure 5) demonstrated that IL-17 was the first cytokine to peak (at 12 hours after arthritis induction), followed by IL-6, TNFα, IL-1β, and IFNγ at 24 hours after the challenge. IL-12 appeared later, peaking on day 4, although the results were not statistically significant. All of the analyzed chemokines (CXCL2, CXCL10, and CCL2) peaked at 24 hours after arthritis induction. CXCL10 remained elevated throughout the response, whereas CCL2 and CXCL2 had decreased to control levels again by day 14 after arthritis induction. In addition, RANKL, a marker of osteoclast differentiation and activation, was elevated from 24 hours after arthritis induction, peaked on day 4, and had fallen to control levels again by day 14.

### Effects of TNFα blockade and dexamethasone treatment in the DTH-arthritis model

**Figure 6:**
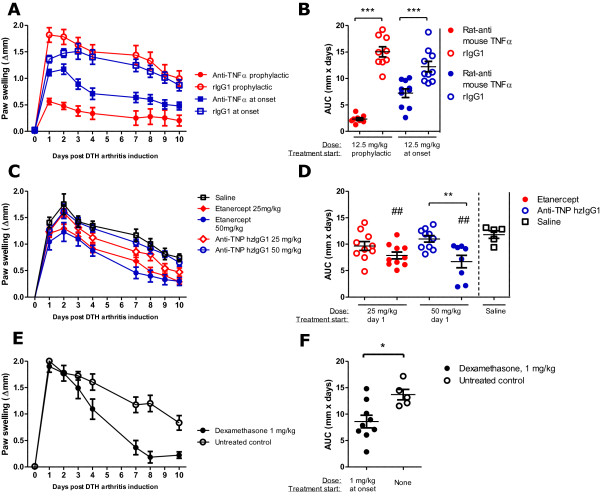
**Paw swelling response in delayed-type hypersensitivity (DTH) arthritis following treatment with tumor necrosis factor alpha (TNFα)-blocking biologics or dexamethasone**. **(a, b) **Rat anti-mouse TNFα mAb (anti-TNFα) was administered from the time of immunization (prophylactic), and control groups received rat IgG1 (rIgG1). Mean ± standard error of the mean (SEM) is shown (*n *= 9 to 10 per group). Area under the curve (AUC) was calculated from days 0 to 10 after arthritis induction. Both prophylactic treatment and treatment at onset with anti-TNFα resulted in a significant reduction in the footpad swelling response. **(c, d) **Etanercept, a soluble TNF receptor fused to the Fc part of human IgG, was administered therapeutically (from day 1), and control groups received humanized anti-trinitrophenol (anti-TNP hzIgG1). A vehicle (saline) control group was also included. Mean ± SEM is shown (*n *= 9 to 10 per group). AUC was calculated from days 0 to 10 after arthritis induction. Therapeutic treatment with etanercept resulted in a significant reduction in footpad swelling when administered at 50 mg/kg. Treatment with 25 and 50 mg/kg both resulted in a significant reduction in the footpad swelling response when compared with the saline control group (indicated by ##). **(e, f) **Dexamethasone, a glucocorticoid, was administered daily from time of disease onset, and control groups received no treatment. Mean ± SEM is shown (*n *= 9 to 10 per group). AUC was calculated from days 0 to 10 after arthritis induction. Daily treatment with dexamethasone from time of disease onset significantly reduced the paw swelling response when compared with the untreated control group. The levels of significance are defined as follows: *P ≤ 0.05, **P ≤ 0.01, ***P ≤ 0.001, ^##^*P *≤ 0.01.

**Figure 7:**
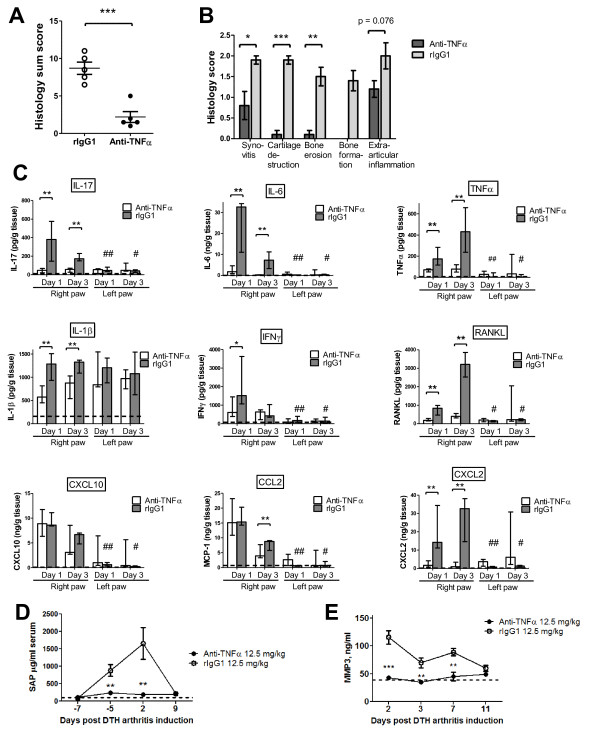
**Effect of treatment with TNFα blocking biologics on histology, systemic and local inflammatory parameters**. **(a, b) **Rat anti-mouse tumor necrosis factor-alpha monoclonal antibody (anti-TNFα) treatment led to a significant reduction in both the histology sum score and the individual parameters of the sum score in comparison with the control group. *P ≤ 0.05, **P ≤ 0.01, ***P ≤ 0.001. **(c) **Local profile of inflammatory mediators following administration of anti-TNFα (*n *= 5 per time point). Median and range are shown. Dotted line represents levels measured in naïve mice (*n *= 5). *P ≤ 0.05, **P ≤ 0.01, ***P ≤ 0.001, compared with value obtained from the mBSA-challenged paws taken from the isotype control group. ^#^*P *≤ 0.05, ^##^*P *≤ 0.01, ^###^*P *≤ 0.001, compared with the value obtained from the contralateral phosphate-buffered saline (PBS)-challenged paws taken from animals receiving identical treatments. **(d) **Serum levels of serum amyloid P component (SAP) in mice given prophylactic treatment with either anti-TNFα or isotype control antibody. Mean ± standard error of the mean (SEM) is shown (*n *= 5). **P ≤ 0.01, compared with the control group. Dotted line represents levels measured in naïve mice (*n *= 5). **(e) **Serum levels the enzyme MMP3 in mice given prophylactic treatment with either anti-TNFα mAb or isotype control antibody (rIgG1). Mean ± SEM is shown (*n *= 5). Dotted line represents levels measured in naïve mice (*n *= 5). **P ≤ 0.01, ***P ≤ 0.001, compared with the control group. CXCL, chemokine (C-X-C motif) ligand; IFNγ, interferon-gamma; IL, interleukin; MCP-1, monocyte chemo attractant protein 1; MMP3, matrix metalloproteinase 3; RANKL, receptor activator of nuclear factor kappa-B ligand; SAP, serum amyloid P component.

As the DTH-arthritis model was newly established, it was desirable to investigate whether the model responded to treatment with biologics or small-molecule anti-inflammatory compounds used in the treatment of RA. Two methods of blocking TNFα were tested: etanercept, a soluble TNF receptor fused to the Fc part of human IgG, and rat anti-mouse TNFα mAb (anti-TNFα). Anti-TNFα was administered prophylactically from the time of immunization and as treatment at onset from the time of mBSA challenge and in both instances was able to significantly reduce the footpad swelling response (Figure 6a, b). Etanercept was administered therapeutically in doses of 25 and 50 mg/kg, one day after disease onset when severe paw swelling was observed, and was able to significantly reduce the footpad swelling response when 50 mg/kg was given (Figure 6c, d). Treatment with 25 and 50 mg/kg etanercept both resulted in a significant reduction in the footpad swelling response in comparison with the saline control group (Figure 6b). The glucocorticoid dexamethasone was administered in doses of 1 mg/kg from the time of disease onset and was able to significantly reduce the paw swelling response in comparison with the untreated control group (Figure 6e, f). These data demonstrate that DTH arthritis responds to prophylactic and therapeutic intervention with TNF-blocking biologics and dexamethasone. To demonstrate the effect of treatment on multiple readouts, we performed a histopathological analysis on mBSA-challenged paws from mice treated with anti-TNFα. The results showed that treatment with anti-TNFα led to both a reduced histology sum score (Figure 7a) and a reduction in all of the individual parameters analyzed (Figure 7b). We also performed an analysis of the production of inflammatory mediators in the mBSA-challenged and PBS-challenged hind paws on days 1 and 3 after DTH-arthritis induction on mice given prophylactic treatment with anti-TNFα mAb or the corresponding isotype control antibody (Figure 7c). The results demonstrated that prophylactic administration of anti-TNFα mAb led to a decrease in production of a range of inflammatory mediators in comparison with mice receiving the corresponding isotype control antibody. In addition, there was a significant difference between the levels in the mBSA-challenged and contralateral PBS-challenged paw for the majority of the analyzed inflammatory mediators (Figure 7c), demonstrating the usefulness of the contralateral PBS-challenged hind paw as an intra-animal control. IL-1β was an exception, however, as the levels of this cytokine measured in the contralateral PBS-challenged paw were no different from the levels measured in the mBSA-challenged paw. This could be due to an effect of the anti-CII cocktail alone on the production of this cytokine. We also measured serum levels of SAP (Figure 7d) and MMP3 (Figure 7e) in mice receiving anti-TNFα mAb. We found the serum levels of these to be significantly lower in mice given anti-TNFα than in mice given the isotype control antibody. Taken together, these data demonstrate that prophylactic treatment with anti-TNFα mAb led to significant decreases in the histopathological score, the production of a range of inflammatory mediators locally in the mBSA-challenged paw, and serum levels of SAP and total MMP3. In addition, the data strengthen the value of using the contralateral PBS-challenged paw as an intra-animal control, as there is a significantly lower production of a range of inflammatory cytokines and chemokines in this paw compared with the mBSA-challenged paw.

## Discussion

In this study, we have established and characterized a novel arthritis model, the DTH-arthritis model, in female C57BL/6J mice. The model has been described in male BALB/c mice [18]. We found that administering CII between the immunization and challenge steps of an mBSA-induced DTH reaction led to a robust T cell-dependent arthritis of the mBSA-challenged paw as reported by Tanaka and colleagues. We demonstrated that paw swelling persisted for at least 28 days. Pronounced involvement of neutrophils and macrophages was shown, findings supported by the observed local profile of inflammatory mediators. The increase in local IL-17 production suggested an involvement of T helper 17 (Th17) cells, a T-cell subset associated with strong inflammatory responses and autoimmunity [19,20]. However, the very early peak production of this cytokine may also point toward the involvement of mast cells (a cell type also observed in the inflamed joint of patients with human RA [21]), where they have been shown to produce IL-17 [22]. Mast cells have been found to play a role in other murine models of arthritis [21], and the presence and role of mast cells in DTH arthritis are currently under investigation. Histological evaluation of the inflamed paws in the present study showed synovitis, pannus formation, synovial hyperplasia, and bone and cartilage destruction, reminiscent of CIA [23,24] and human RA [25,26]. An increase in osteoclast number was also observed and this coincided with the onset of bone erosion. This finding was further supported by the increased production of RANKL locally in the arthritic paw, which coincided with the increase in TRAP-positive cells on day 4 after arthritis induction. RANKL is a TNF superfamily member expressed by osteoblasts, activated T cells, and synovial fibroblast-like cells. RANKL exists in both a soluble and a membrane-bound form and is an essential stimulatory factor for recruitment, differentiation, and activation of osteoclasts. RANKL binds to its receptor RANK, which is present on the surface of osteoclast precursors and mature osteoclasts [27], and the absence of RANKL prevents bone erosion in murine arthritis [28]. In addition, the cytokines TNFα, IL-6, IL-1, and IL-17, which are elevated early after onset of the inflammatory response in the present study, are known to lead to an increase in RANKL expression [27], and these findings provide evidence that the increased osteoclast activation observed is a result of the inflammatory response in the affected joint. New bone formation was also observed, albeit in an aberrant pattern in the form of osteophytes adjacent to the affected joints. This finding suggests that there is a disturbance in the overall process of bone turnover in DTH arthritis; this disturbance may give an opportunity to study processes of new bone formation in inflammatory arthritis.

Onset and disease course of DTH arthritis were synchronized, incidence was 100% or close to it, and variation was low. These are very desirable features in an animal model, as all animals are exposed to disease and any treatment applied for an equal period of time. The fact that the arthritis affects only one paw allows the study of a more severe paw inflammation without severely compromising animal welfare; in other established models of arthritis, the humane endpoints set in place to limit animal suffering are more frequently reached, as the animals display severe inflammation in multiple paws. In addition, the phenotype of DTH arthritis makes the model very suitable for the application of *in vivo *imaging techniques to study inflammation and bone destruction because of the presence of an intra-animal contralateral control paw and the low intra-group variability. Although the arthritic phenotype is isolated to one paw, the model also displays a systemic component, illustrated by the increase in serum levels of SAP, IL-6, and total MMP3. MMP3 is notable in this situation, as studies have shown the serum levels of this enzyme to be a biomarker of disease severity in RA [29]. The fact that the arthritic phenotype is induced in only one paw, however, also represents a limitation of the DTH-arthritis model by phenotypically removing the animal model from the human condition that it seeks to mimic: RA, which usually affects the joints of multiple extremities in a symmetrical pattern. Whether self-tolerance is broken in DTH arthritis was not investigated in the current study but remains an intriguing possibility. Breach of self-tolerance to joint antigens has been observed in a model of mono-arthritis, a model also induced by an inflammatory response to an antigen unrelated to the joint [30], the hypothesis being that joint auto-antigens are exposed to or released from the inflamed joint during the inflammatory response following antigen challenge. Thus, it remains a possibility that self-tolerance to joint-derived auto-antigens is broken in DTH arthritis.

The role played by anti-CII antibodies in disease induction is still not fully clear, but we hypothesize, on the basis of our results and available literature, that anti-CII antibodies play a role similar to what is observed in CAIA [13-15], namely that they activate complement and cells of the innate immune system through Fc receptors and that these events drive the joint pathology once the challenge with mBSA has initiated the T cell-dependent inflammatory response. The extent to which the anti-CII mAbs localize to the joint space and coat the cartilage surface immediately after administration remains unclear. It is clear, however, that there is no immediate effect of the systemic administration of anti-CII and that the inflammatory milieu of the DTH reaction is necessary to initiate the events leading to joint pathology in DTH arthritis. Anti-CII antibodies have been shown not to localize to the joints without the presence of an exogenous trigger [31]. However, a different study demonstrated that anti-CII antibodies coated the cartilage surface of joints without any previous stimuli to increase vasopermeability [32]. Thus, it is still unclear whether the anti-CII localizes to the joint space only after antigen challenge, due to the local increase in vasopermeability caused by inflammatory mediators and the effects of anti-antigen-antigen immune complexes on complement and FcR activation as some findings indicate [31], or whether it localizes there immediately following administration, at which time it is not pathogenic without further inflammatory stimuli. These questions are currently being addressed by means of *in vivo *imaging techniques. We did, however, measure equal levels of IL-1β in the mBSA-challenged and the PBS-challenged paws on days 1 and 3 after DTH-arthritis induction, and these measurements could point toward a direct effect of the anti-CII antibodies on the production of this cytokine.

We chose to qualify the model with rat anti-mouse TNFα and etanercept (TNFRIg), which is approved for use in the clinic to treat patients with RA. Both agents are efficacious in several other experimental arthritis models [33-35]. Taken together, the results from the two treatment studies demonstrated that DTH arthritis can be suppressed by systemic administration of TNFα-blocking biologics whether administration was prophylactic, at the time of DTH-arthritis induction, or therapeutic from day 1 after DTH-arthritis induction, when paw swelling had reached peak level. In addition, blocking TNFα leads to effects on multiple readouts, both systemic and local, including serum biomarkers also useful as biomarkers in RA: MMP3 and acute-phase proteins. Also, most of the arthritic changes observed histopathologically were prevented when TNFα was blocked in a prophylactic manner. Together, these results suggest that the DTH-arthritis model of experimental arthritis could have some degree of predictive validity with regard to the therapeutic efficacy of biologics for the treatment of RA. In addition, the efficacy of blocking TNFα suggests that DTH arthritis indeed shares immunological pathways with human RA.

## Conclusions

In this study, we have shown that it is possible to elicit a robust arthritis in C57BL/6 mice with synchronized onset and progression and high incidence displaying severe bone and cartilage destruction, something that presents difficulties in the CAIA [15] and CIA [36,37] models when using the C57BL/6 strain. In these models, the disease responses and incidence frequencies observable in C57BL/6 mice are much lower and more variable. We have verified that DTH-arthritis induction is dependent on CD4^+ ^T cells and anti-CII antibody-mediated tissue damage of the joint. Moreover, we have shown the arthritic pathology by histology and found that it is characterized by synovitis, hyperplasia of the synovial membrane, pannus formation, enhanced osteoclast activity, bone erosion and remodeling, and cartilage destruction, features also observed in human RA [25,26]. In addition, inflammation of the affected joints is governed by a massive neutrophil and macrophage infiltration, verified by flow cytometry, histopathology, and analysis of the involved cytokines and chemokines, which also demonstrates the involvement of T cells. We have shown that disease can be reduced by both preventive and therapeutic treatment with TNFα-blocking biologics. Importantly, therapeutic intervention was effective when initiated at peak of disease (day 1 after the challenge). The possibility of robust arthritis induction in C57BL/6 mice allows studies that use genetically modified mice on a C57BL/6 background, permitting accurate comparison of disease in wild-type and genetically modified mice, and this represents a major advantage. Importantly, because disease onset is pre-defined and simultaneous, the start of a treatment plan can be standardized both within and across treatment groups. In addition, all animals are exposed to disease and treatment for exactly the same amounts of time. When seen from a pharmacological perspective, this represents an improvement compared with the CIA model, the current gold-standard arthritis model in the mouse, which has a lower and more variable frequency of disease incidence and a non-synchronized onset [38]. In addition, the DTH-arthritis model is run in female mice, which are much less prone to grouping stress than male mice and thus are easier to house. In summary, we believe that the DTH-arthritis model may be highly useful in the pre-clinical screening of potential protein-based drugs targeting RA, since our data demonstrate that drugs approved for RA treatment have an effect in the model. In addition, the model is highly suited for the application of *in vivo *imaging techniques for studying inflammation and bone remodeling and yields reliable and reproducible data, and this strengthens its potential. Still, additional work is needed to further characterize the immunological mechanisms underlying DTH arthritis and the degree to which they can be aligned to RA pathogenesis.

## Abbreviations

anti-CII: anti-type II collagen monoclonal antibodies; CAIA: collagen antibody-induced arthritis; CFA: complete Freund's adjuvant; CIA: collagen-induced arthritis; CII: type II collagen; CXCL: chemokine (C-X-C motif) ligand; DTH: delayed-type hypersensitivity; H&E: hematoxylin and eosin; IFNγ: interferon-gamma; IL: interleukin; mAb: monoclonal antibody; mBSA: methylated bovine serum albumin; MMP3: matrix metalloproteinase 3; PBS: phosphate-buffered saline; RA: rheumatoid arthritis; RANKL: receptor activator of nuclear factor kappa-B ligand; SAP: serum amyloid P component; SEM: standard error of the mean; TNF: tumor necrosis factor; TNFR: tumor necrosis factor receptor; TRAP: tartrate-resistant acid phosphatase.

## Competing interests

The authors declare that they have no competing interests.

## Authors' contributions

SMA designed and carried out all studies and analyses in the paper and drafted the manuscript. PAU developed the histological scoring system and helped draft the manuscript. PHK provided invaluable assistance with the histological evaluations and helped draft the manuscript. AN and CH conceived of the study and participated in its design and coordination and helped draft the manuscript. HM provided intellectual support and helped draft the manuscript. All authors read and approved the final manuscript.
